# Diseases of the Throat, Nose, and Ear

**Published:** 1892-06

**Authors:** 


*Diseases of the Throat, Nose, and Ear.* By P. McBRIDE, M.D., F.R.C.P. Ed. Pp. xvi., 640. Edinburgh: YOUNG J. PENTLAND. 1892.

There is no doubt that this book will take a permanent place as a standard work on throat, nose, and ear disease. The author deals especially fully with the diagnosis and treatment of ear disease, and nearly three hundred pages are devoted to this section. Of this we are very glad, as we believe that the man who can examine and treat pathological conditions of the nose and naso-pharynx is more successful in treating ear cases than the pure aurist, to whom posterior rhinoscopy reveals little or nothing of naso-pharyngeal disease. We have read with much pleasure the valuable practical hints that are given as to syringing and drying the ear; and we agree with the author that Schwartze's caustic treatment is apt to be most mischievous in small perforations. It is also satisfactory to find that the author deprecates operations on the anterior nares and pharynx for the relief of deafness, unless surgical interference is necessary on rhinological and laryngological grounds. Some distinguished specialists are always ready to mutilate their patients' noses by removing spurs, and even pieces of turbinated bones, and a note of warning is useful.

Dr. McBride is a firm believer in the virtues of pilocarpine for labyrinthine disease, and we have seen it do much good, although scepticism is freely expressed by some. "Necrosing ethmoiditis" does not find a place in the index, and the author is evidently not a disciple of Woakes. The influence of influenza on the larynx and ear is well described, and the persistence of pain after discharge from the ear has well begun must now be looked on as *the* symptom of influenza suppurative ear disease. We quite agree with the author that slight hypertrophy of the lingual tonsil gives rise to symptoms in anæmic or hysterical women, who ought to become acquainted with iron in the form of pills rather than as wire made red hot. The reckless way in which the electric cautery is used in these and kindred troubles is most mischievous.

The book abounds in practical hints for examining and treating patients; and although some of the illustrations are not quite so well drawn and coloured as we have seen them in books dealing with throat and nose disease, yet we consider that it is a most valuable treatise, reflecting great credit on author and publisher.

